# Deep sequencing reveals the first fabavirus infecting peach

**DOI:** 10.1038/s41598-017-11743-7

**Published:** 2017-09-12

**Authors:** Yan He, Li Cai, Lingling Zhou, Zuokun Yang, Ni Hong, Guoping Wang, Shifang Li, Wenxing Xu

**Affiliations:** 1 State Key Laboratory of Agricultural Microbiology, Wuhan, Hubei 430070 P.R. China; 2 College of Plant Science and Technology, Huazhong Agricultural University, Wuhan, Hubei 430070 P.R. China; 3 Key Lab of Plant Pathology of Hubei Province, Wuhan, Hubei 430070 P.R. China; 4 State Key Laboratory of Biology of Plant Diseases and Insect Pests, Institute of Plant Protection, Chinese Academy of Agricultural Sciences, Beijing, 100094 P.R. China

## Abstract

A disease causing smaller and cracked fruit affects peach [*Prunus persica* (L.) Batsch], resulting in significant decreases in yield and quality. In this study, peach tree leaves showing typical symptoms were subjected to deep sequencing of small RNAs for a complete survey of presumed causal viral pathogens. The results revealed two known viroids (*Hop stunt viroid* and *Peach latent mosaic viroid*), two known viruses (*Apple chlorotic leaf spot trichovirus* and *Plum bark necrosis stem pitting-associated virus*) and a novel virus provisionally named Peach leaf pitting-associated virus (PLPaV). Phylogenetic analysis based on RNA-dependent RNA polymerase placed PLPaV into a separate cluster under the genus *Fabavirus* in the family *Secoviridae*. The genome consists of two positive-sense single-stranded RNAs, i.e., RNA1 [6,357 nt, with a 48-nt poly(A) tail] and RNA2 [3,862 nt, with a 25-nt poly(A) containing two cytosines]. Biological tests of GF305 peach indicator seedlings indicated a leaf-pitting symptom rather than the smaller and cracked fruit symptoms related to virus and viroid infection. To our knowledge, this is the first report of a fabavirus infecting peach. PLPaV presents several new molecular and biological features that are absent in other fabaviruses, contributing to an overall better understanding of fabaviruses.

## Introduction

Peach [*Prunus persica* (L.) Batsch] has been cultivated for more than 3000 years in China, which is considered to be the original and evolutionary center of peach^1^. Peach is revered as a delicious and healthy summer fruit in most temperate regions of the world and is cultivated in more than 80 countries and regions. Peach is a major commercial fruit crop in the top five producing countries: China, Italy, Spain, the USA, and Greece. In addition, viral agents are economically important, particularly when they affect fruit quality or induce severe tree decline, and are most likely also involved in other diseases with uncertain etiology^1^.

Abnormal symptoms, including smaller and cracked fruits, are easily observed in peach orchards in China. In most cases, these symptoms are considered to be induced by climate, nutritional deficiency, cultivation practices, or abiotic factors. Although symptoms typically appear in a uniform manner for all fruits on the same tree, in China, abnormal symptoms have been observed in a non-uniform manner, whereby only some fruits on the same tree are affected. The pathological agent(s) responsible for these symptoms remain unknown. As the symptoms have not been associated with fungal or bacterial infection, viral pathogens are considered potential etiological agents responsible for the disease.

In this study, we applied deep sequencing to characterize viruses and viroids associated with the peach disease characterized by smaller and cracked fruits. The data revealed the presence of two known viroids (*Hop stunt viroid*, HSVd, and *Peach latent mosaic viroid*, PLMVd), two known viruses (*Apple chlorotic leaf spot trichovirus*, ACLSV, and *Plum bark necrosis stem pitting-associated virus*, PBNSPaV) and a novel fabavirus (provisionally named *Peach leaf pitting-associated virus*, PLPaV). Further biological assays of the co-infecting viruses and viroids using GF305 peach indicator seedlings revealed a novel leaf-pitting symptom rather than the smaller and cracked fruit symptoms related to the viral agents, which is most likely associated with PLPaV.

## Results

### Raw deep-sequencing data

**Figure 1:**
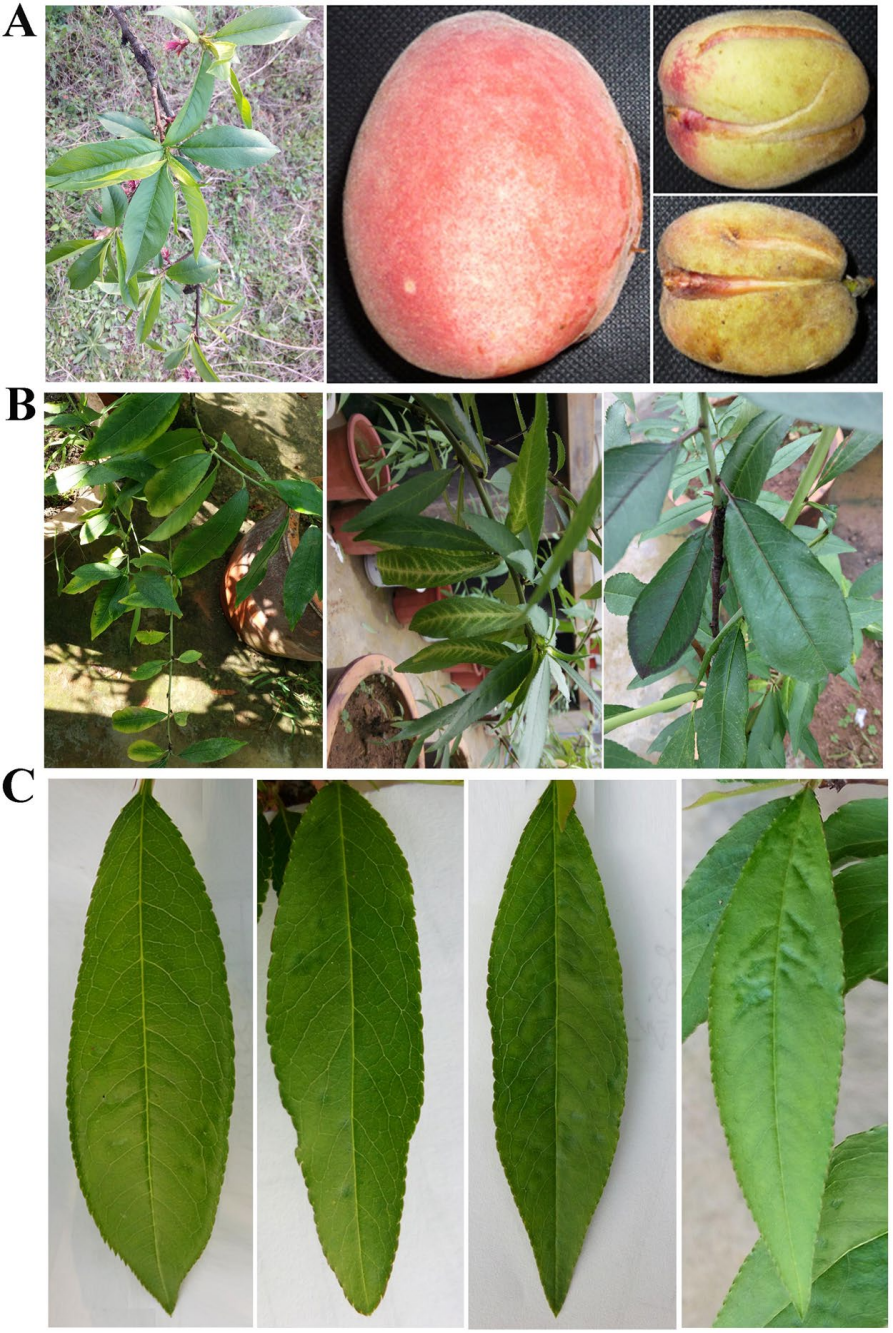
Symptoms of smaller and cracked fruits on the peach tree (sample XJ-6) used for deep sequencing and the induced symptoms on GF305 peach indicator seedlings. (**A**) Asymptomatic leaves (left panel), healthy fruits (middle) and smaller and cracked fruits (right) on XJ-6 peach tree. (**B**) Variable symptoms including chlorosis along leaf edges (left), calico coloring along leaf veins (middle), and dark violet coloring of leaf petioles, veins or edges (right) in a GF305 seedling (no. 1 to no. 3, respectively). (**C**) Leaf pitting symptoms observed in the three inoculated GF305 seedlings.

Leaves were collected from a symptomatic peach tree (sample XJ-6) displaying typical smaller and cracked fruit symptoms (Fig. 1). The sample was characterized as having some fruits of a smaller size that were cracked (3.2–3.8 × 4.4–5.0 cm) compared with the remaining fruits (5.7–6.7 × 7.7–8.4 cm) on the same tree, but the leaves showed no symptoms, as assessed by a three-year survey. A library prepared from RNA obtained from the small leaves (sRNA) was sequenced using the Solexa-Illumina platform, and 5,829, 462 clean reads were obtained. The majority of reads were 16 to 30 nt in length, with three peaks of 16, 21 and 24 nt (Fig. S1A). Analysis of the 5′-terminal nucleotide revealed a clear bias depending on size, excluding the 20- and 30-nt-sized sRNAs: A (39.8%, 62.3%, 48.7%, 39.8% and 65.4%) for sRNAs of 19, 24, 25, 28 and 29 nt; U (36.3% and 43.7%) for 21 to 23 nt; G (78.6%) for 16 nt; and C (37.8% and 36.1%) for 17 and 26 nt (Fig. S1B).

### Identification and characterization of a novel fabavirus in peach trees exhibiting smaller and cracked fruit symptoms

**Figure 2:**
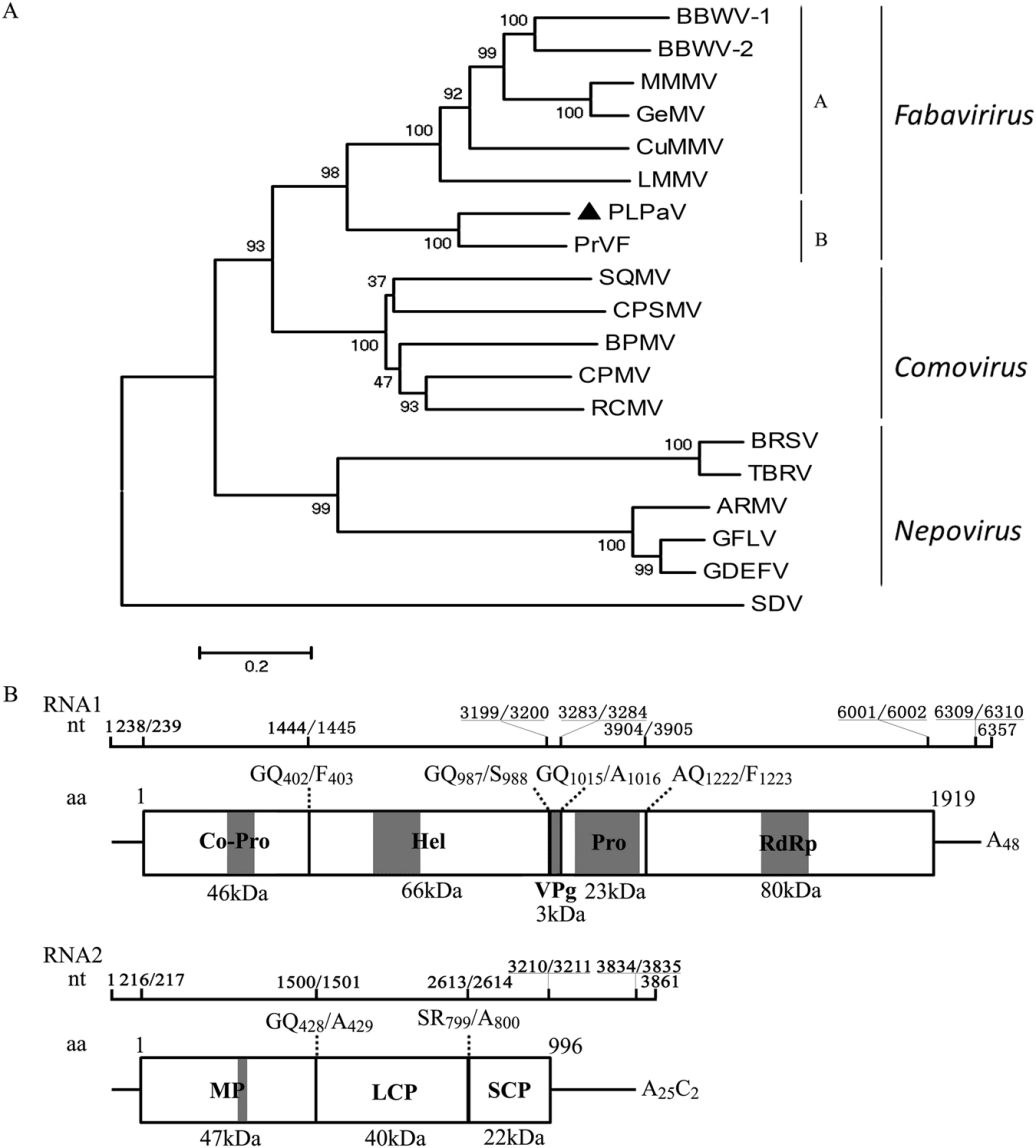
The phylogenetic tree based on the amino acid sequences of the RdRp gene of typical and selected members of *Comovirirdae* and the genome organization of Peach leaf pitting-associated virus (PLPaV). (**A**) Evolutionary history was inferred using the neighbor-joining (NJ) method. The tree is drawn to scale, with branch lengths in the same units as those of the evolutionary distances used to infer the phylogenetic tree. The evolutionary distances were computed using the JTT matrix-based method and are presented in units of the number of amino acid substitutions per site. GenBank accession numbers, genera and acronyms of the involved viruses are listed in Table S3. (**B**) Genome organization of PLPaV, showing the relative position of the open reading frames (ORFs) and their expressed products. Vertical lines through the long rectangles indicate putative sites of polyprotein cleavage. Calculated values of Mr and positions of mature proteins are indicated. Co-pro, cofactor required for proteinase; Hel, putative helicase; VPg, genome-linked protein; Pro, proteinase; RdRp, RNA-dependent RNA polymerase; MP, movement protein; LCP, large coat protein; and SCP, small coat protein. Conserved motifs in each protein are indicated by shading.

*De novo* assembly of sRNAs generated five sequence contigs, with lengths ranging from 284 to 513 nt and high amino acid similarity with members of subfamily *Comovirinae*, family *Secoviridae*. To obtain the complete genomic sequence of what appeared to be a novel member of this family, overlapping reverse transcription polymerase chain reaction (RT-PCR) products covering the entire genome were generated using primers based on the above contigs (Table S1; Fig. S2). The sequences obtained by Sanger sequencing were in agreement with those generated from sRNA assembly. After sequencing the 5′- and 3′-terminal regions, the complete bipartite genomes together with their poly(A) tails were determined to be 6,357 nt [6,309 nt without poly(A)] and 3,861 nt [3,834 nt without poly(A)] for RNA1 and RNA2, respectively (GenBank Accession Nos. KY867750 and KY867751). A BLASTn search of the full-length nucleotide sequences indicated no detectable similarity with known viruses in the NCBI database. A BLASTp search revealed that the deduced proteins (see below) encoded by both RNAs show the highest similarities to both polyproteins of *Prunus virus F* (PrVF) recently isolated from sweet cherry (ANH71248 and ANH71253, coverage 89% and 72%, e-values 0.0 and 0.0, and identities 66% and 46%, respectively), with 46% identity (coverage 95%, e-value 4.0e^−168^) for the putative coat proteins (CPs) and 49% identity (coverage 89%, e-value 2.0e^−114^) for the putative proteinase cofactor (Co-Pro). Species demarcation criteria for the family *Secoviridae* (i.e., amino acid sequences of CP and Co-Pro regions with less than 75% and 80% identity, respectively)^2^ suggest that RNA1 and RNA2 are genomic RNAs of a novel virus, provisionally named Peach leaf pitting-associated virus (PLPaV, designated isolate PLPaV-XJ-6) based on the related symptoms (see below). Phylogenetic comparison of the amino acid sequence of the RNA-dependent RNA polymerase (RdRp) of PLPaV with those of selected members of the family *Secoviridae* positioned PLPaV together with PrVF into a separate cluster in the genus *Fabavirus* and in an outgroup from other members (Fig. 2A). These findings support that PLPaV and PrVF are phylogenetically distantly related to viruses isolated from non-*Rosaceae* plants. Here, we propose placing subgroups A and B in the genus *Fabavirus* (Fig. 2A), which accommodates fabaviruses of *Rosaceae* and non-*Rosaceae* plants, respectively. Correspondingly, both PLPaV and PrVF share significantly lower similarity with other fabaviruses (46.3–48.7%) compared with other fabaviruses (54.3–68.6%).

### Genomic organization and conserved motifs of PLPaV RNAs

PLPaV shares a similar genomic organization with known fabaviruses (exemplified by the genomic organization of RNA1 shown in Fig. S3A). The 5′-untranslated region (5′-UTR) of PLPaV RNA1 is 238 nt, rich in U (21.43% A, 42.02% U, 14.71% G, 21.85% C) and A + U (63.45%), and contains the repeated motif (CAGCUUUC) from positions 20 to 28 and from 53 to 61. RNA1 was concluded to contain a single, long open reading frame (ORF) starting with an initiation codon (AUG) at positions 239 through 241 and terminating at a termination codon (UAA) at positions 6001 through 6003, encoding a polyprotein of 1,919 amino acids with a calculated molecular weight (MW) of 217 kDa. Putative sites of proteolytic cleavage were identified at the dipeptides Q_402_/F_403_, Q_987_/S_988_, Q_1015_/A_1016_, and Q_1222_/F_1223_, resulting in proteins corresponding to Co-Pro, helicase (Hel), genome-linked protein (VPg), proteinase (Pro) and RdRp (Fig. 2B), respectively. The deduced Co-Pro and Hel proteins possess motifs similar to those conserved in other fabaviruses and positive-strand RNA viruses, respectively (Fig. S3B and C)^3–6^. The deduced VPg protein harbors a motif similar to that (E/DX3YX3NX4–5R) conserved in VPg of the family *Comoviridae*^7,8^, with a change of the R residue at position 914 to a K residue (Fig. S3D). The remaining hypothesized proteins also contain conserved motifs observed in other fabaviruses or double-stranded RNA viruses (Fig. S3E and F)^4,6,9^. The 3′-UTR consists of 308 nt rich in U (19.81% A, 39.61% U, 21.75% G, 18.83% C) and A + U (59.42%), with a 48-nt-long poly(A) tail.

The 5′-UTR of PLPaV RNA2 is 216 nt long, rich in U (23.50% A, 42.86% U, 12.44% G, 21.20% C) and A + U (66.36%), and contains the same repeated motif (CAGCUUUC) found in RNA1 at positions 20 through 27. The complete nucleotide sequence of PLPaV RNA2 also contains a single long ORF, which is initiated at position 217 and terminates at position 3210 (Fig. 2B) and encodes a polyprotein of 996 aa with a calculated MW of 109 kDa. Putative sites of proteolytic cleavage were identified at dipeptides Q_428_/A_429_ and R_799_/A_800_, resulting in proteins corresponding to movement protein (MP), large coat protein (LCP), and small coat protein (SCP) (Fig. 2B), respectively. The 3′-UTR is 625 nt and rich in U (20.03% A, 39.10% U, 21.79% G, 19.07% C) and A + U (59.13%), with a 25-nt-long poly(A) tail containing two cytosines at positions 3837 and 3848.

**Figure 3:**
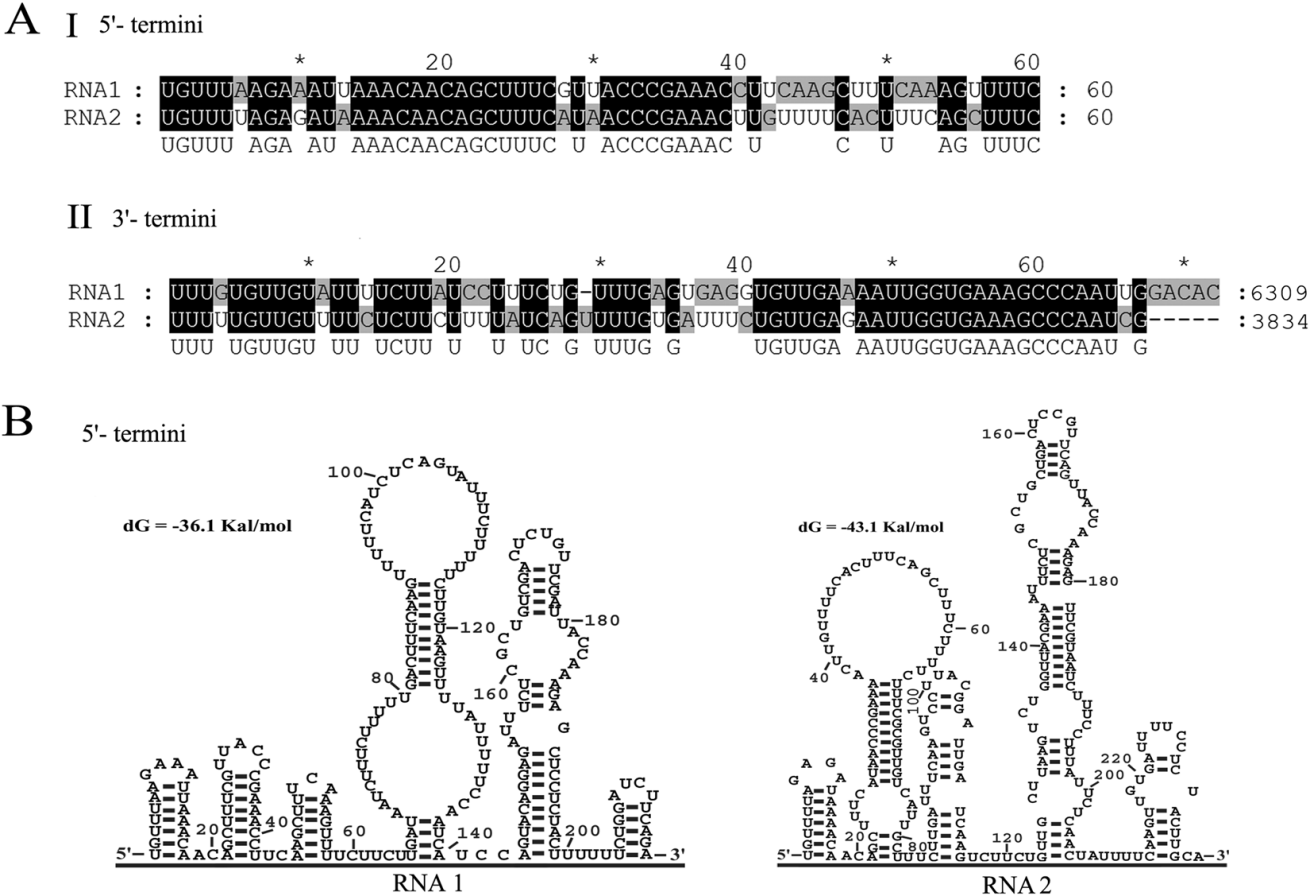
Sequence alignments and predicted secondary structures of the terminal regions of RNA1 and 2 of PLPaV. (**A**) Conserved sequences of the 5′ terminus (I) and 3′ terminus (II) of the RNA1 and -2 of PLPaV, respectively. Black, gray, and light gray backgrounds, nucleotide identities of no less than 100%, 80%, and 60%, respectively. (**B**) Secondary structures proposed for 5′-UTRs of RNA1 and -2 of PLPaV with the lowest energies (http://mfold.rna.albany.edu/?qDINAMelt/Quickfold).

The 5′-UTRs of the RNAs share high identity, of 60.7%, and 43 nt are identical, compared with only 60 nt at each 5′ end (Fig. 3A). Moreover, the 5′-UTRs of both RNAs are predicted to form compact stem/loop structures, among which the first 20 nucleotides of both strands may form a stem/loop structure similar to hairpin I (Fig. 3B**)**. The 3′-UTRs of the RNAs are 78.9% identical, and the consensus sequence (UAGU or UAUGU), which plays a role in transcriptional termination of some genes and in polyadenylation of their transcripts in yeast^10^, was found with a considerably high frequency at six to eight positions upstream of the poly(A) tail of both UTRs (Fig. 3A). In contrast, the polyadenylation signal (AAUAAA) for most eukaryotic mRNAs^11^ was absent in the UTRs.

### Distribution of small interference RNAs (siRNAs) along the PLPaV genome

**Figure 4:**
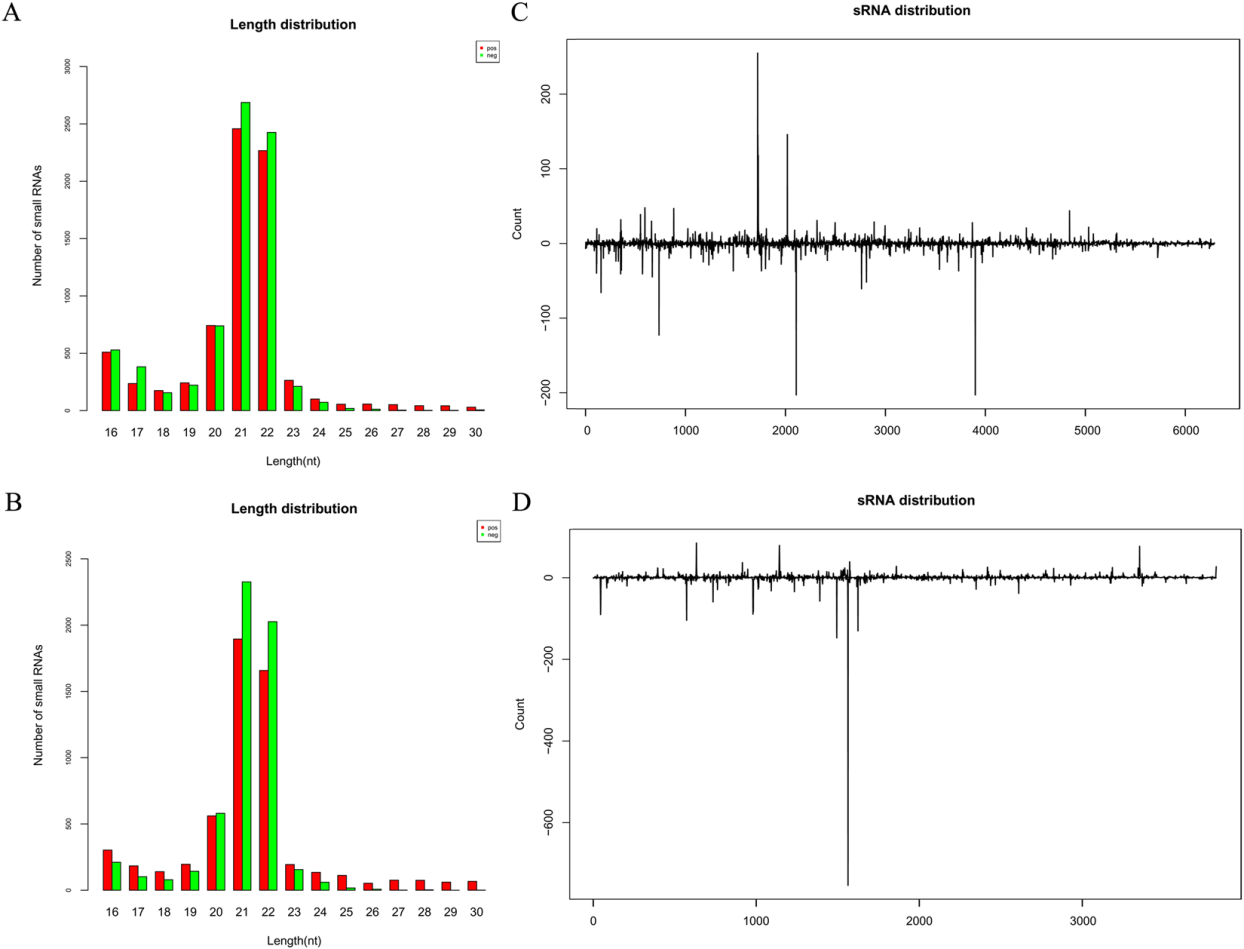
Size distribution of small RNAs (sRNAs). (**A**) Bar graph showing the size distributions of sRNAs in the library prepared from XJ-6 peach leaves. (**B**) Distribution of all size classes (16–30 nucleotides) of viral small interference RNAs (vsiRNAs) captured by high-throughput sequencing along the PLPaV genome.

The majority of siRNA reads were 16–23 nt in length, with two dominant peaks at 21 and 22 nt for both strands (Fig. 4A and B). Alignments of the siRNA and PLPaV sequences showed that the former completely cover the PLPaV genome (Fig. 4C and D). The frequency and distribution of siRNA coverage was continuously but heterogeneously distributed throughout PLPaV RNA1 and RNA2, whereas a slightly decreased accumulation at the 3′′ terminal region of PLPaV RNA1 was observed, regardless of RNA polarity (Fig. 4C and D). Examination of siRNA profiles revealed three and one notable hotspots (more than 200 reads) along the RNA1 and RNA2 strands, respectively (Fig. 4C and D). The hotspot for RNA1 was observed at 1722–1743 (22 nt: UGCAUAUAUUUUCUGUGGCACC) in positive polarity and for RNA2 at 1563–1583 (21 nt: GCGUUUACUGUUCUCAGGUCG) in negative polarity (Fig. 4C and D). The GC contents of the hotspots on the positive and negative strands are 41% and 52%, respectively, clearly lower than those observed in other viruses^12,13^. Moreover, the most prominent peaks of sequence abundance correspond to 21 or 22 nt siRNAs and localize to the same genomic regions, as previously observed for *Sugarcane mosaic virus* (SMV)^14^.

**Figure 5:**
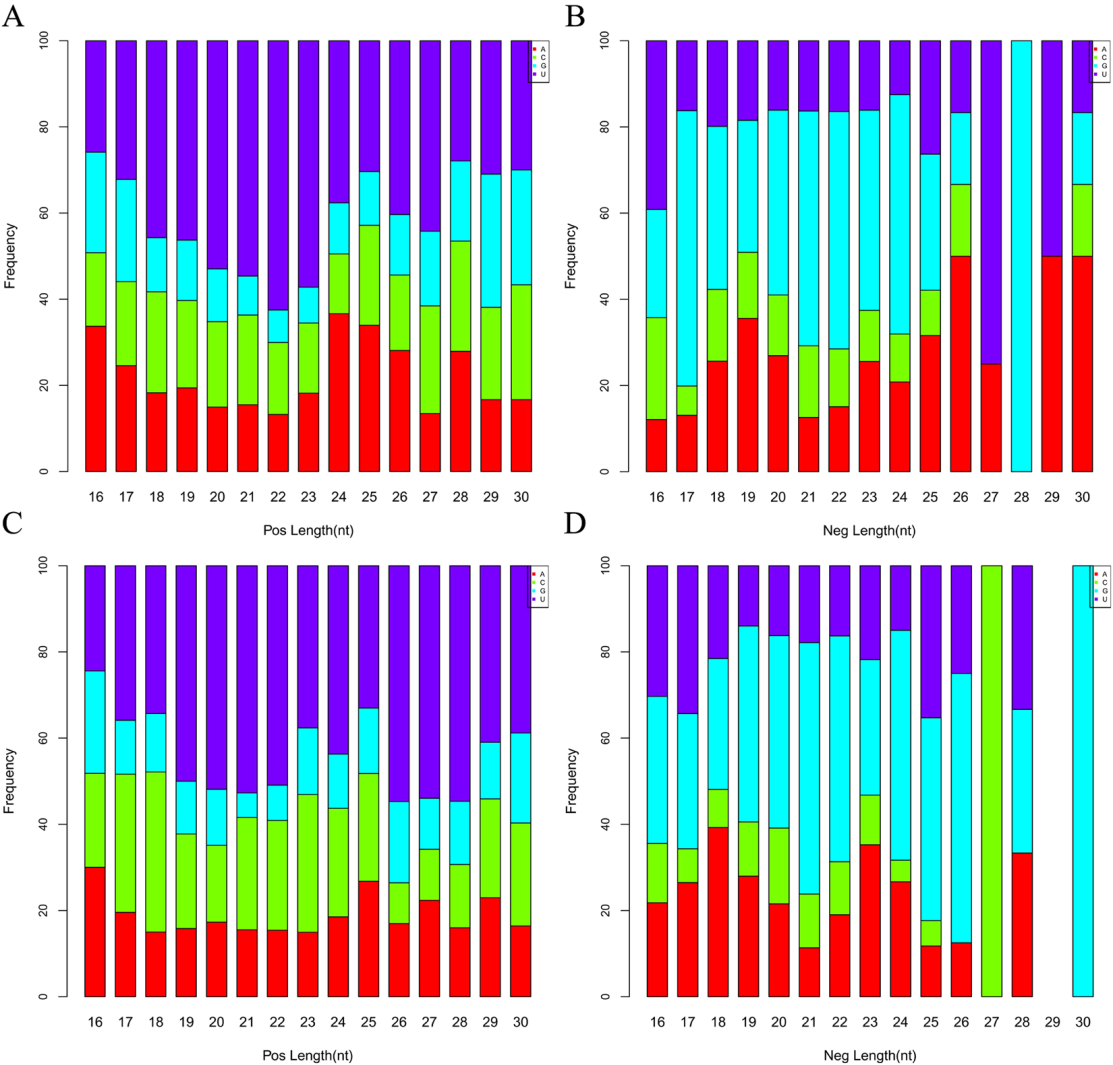
The percentage accumulation histogram for the frequency of the 5′-nucleotide of siRNAs. (**A**–**D**) Frequency of the 5′-nucleotide of siRNA derived from the plus (**A**,**C**) and minus (**B**,**D**) strands of RNA1 (**A**,**B**) and RNA2 (**C**,**D**) of PLPaV, respectively.

Analysis of the 5′-terminal nucleotide of the siRNAs revealed an obvious bias depending on the polarity and size, and it is worth noting that a completely dominant G (100%) was found for 28 and 30 nt siRNAs in the negative-polarity strands of both RNAs. In addition, C was completely dominant (100%) for 27 nt siRNAs but absent for 26, 28 and 30 nt siRNAs and underrepresented (5.9–17.6%) for the others in the negative-polarity strand of RNA2 (Fig. 5), whereas no 29-nt siRNAs were detected in the negative-polarity strand of RNA2 (Fig. 5).

### Identification of co-infecting viroids and viruses in the deep-sequencing sample

BLASTn searches of the sequence contigs generated from *de novo* assembly of the sRNAs also revealed the complete genomes of two reported viroids (HSVd and PLMVd) and the partial genomes of two known viruses (ACLSV and PBNSPaV), which were designated as isolates HSVd-XJ-6, PLMVd-XJ-6, ACLSV-XJ-6 and PBNSPaV-XJ-6, respectively. Leaves were collected from the peach tree and used for deep sequencing and RT-PCR identification of all co-infecting viroids and viruses using the primers listed in Table S2. Clear target bands with high concentrations of the expected sizes were obtained for all viroids and viruses only from the deep-sequencing sample (XJ-6) and the positive control samples (GF305 peach previously inoculated with PLMVd; citrus infected with HSVd; pear infected with ACLSV; peach infected with PBNSPaV), and no bands with similarity to the targets were amplified from the negative controls (GF305 peach seedlings without inoculation) (Fig. S4). Further cloning and sequencing of the target bands confirmed co-infection of viroids and viruses. The full-length HSVd-XJ-6 RNA is 297 nt in length, with the same nucleotide sequence as that of an isolate from plum *(Prunus salicina* L.) in Korea (JQ706343)^15^. The full-length PLMVd-XJ-6 RNA is 337 nt in length, with the same nucleotide sequence with that of an isolate from plum (*P. salicina* L.) in Korea (JX479333)^15^. Twenty contigs ranging from 67 to 193 nt were assembled for ACLSV-XJ-6, which shares the highest identity with genomic RNAs of ACLSV isolate Z1 from peach in China (JN634760, 83% coverage, 97% identity, and 9.0e^−70^ e-value). One hundred and seventy-seven contigs were assembled for PBNSPaV-XJ-6, ranging from 47 to 327 nt, sharing the highest identity with genomic RNAs of the PBNSPaV isolate WH from peach in China (KJ792852, 98% coverage, 99% identity, and 0.0 e-value).

### Related symptoms, host range and incidence

To observe the symptoms related to these co-infecting viroids and viruses, buds of the peach sample (XJ-6) used for deep sequencing were grafted onto three GF305 peach seedlings. After four months, the samples were subjected to RT-PCR index analysis of the co-infecting viroids and viruses using the primers and denaturation temperatures provided in Table S2. The results indicated that PLMVd was able to infect all the grafted GF305 peach seedlings, whereas HSVd, ACLSV and PBNSPaV only infected one or two of them (Fig. S4A and B); identification was further confirmed by performing two independent RT-PCR analyses. It is surprising that PLPaV was not successfully amplified by RT-PCR from any of the graft-inoculated seedlings [confirmed using two different primer pairs (Fa1-F/Fab5′R1R and Fa1-1/Fab5′R1R) targeting part of the HEL gene]. However, PLPaV was amplified by nested PCR amplification (nest-PCR) [using the primers Fa1-F/Fab5′R1R followed by Fa1-F/Fab5′R1Rn or Fa1-1/Fab5′R1R followed by Fa1-1/Fab5′R1Rn]. This result indicates a dramatically decreased titer for PLPaV in the graft-inoculated seedlings, even after a long incubation period (more than one year), compared with the donor sample (Fig. S4C). Conversely, the co-infected viroids and viruses were successfully amplified using RT-PCR (Fig. S4A and B).

In contrast to the healthy appearance of the control seedlings, which were not inoculated, in the following spring and autumn, all GF305 peach seedlings grafted with XJ-6 buds displayed leaf chlorosis symptoms along the leaf edge, which is characteristic of PLMVd infection^16^ (Fig. 1B, the left panel). One inoculated GF305 peach seedling also showed other peculiar symptoms on some leaves in July, including calico coloring along the leaf veins (Fig. 1B, middle panel) and dark violet coloring of the leaf petioles, veins or edges (Fig. 1B, right panel). Additionally, a leaf-pitting symptom characterized by many irregular pits that appeared oily and dark green in color was observed on the leaves of all graft-inoculated seedlings (Fig. 1C). At two years post-inoculation, each of the GF305 seedlings began to bear 3–5 fruits, and no obvious differences in fruit size between the healthy controls and infected seedlings were observed when the fruits ripened. Moreover, no symptoms of cracking were observed in the fruits.

A host range assay was performed by mechanical inoculation of ten plants belonging to four families (*Fabaceae*, *Solanaceae*, *Cucurbitaceae*, and *Chenopodiaceae*), including *Vigna unguiculata*, *Vicia faba*, *Pisum sativum*, *Nicotiana occidentalis*, *Nicotiana benthamiana*, *Solanum lycopersicum*, *Cucurbita moschata*, *Cucumis sativus*, *Chenopodium amaranticolor*, and *Chenopodium quinoa* (Table 1), and indexed by RT-PCR followed by nested-PCR (nRT-PCR) for the presence of PLPaV using the above-described primers (Table S2). The results suggested successful infection at 20 days post-inoculation (dpi) only for *P. sativum* seedlings (4/5), whereas further identification using nRT-PCR revealed more than one seedling positive for the virus for all tested plants, except *N. benthamiana* (Table 1; Fig. S5B). To eliminate technical errors that might have resulted in a failure of PLPaV to infect *N. benthamiana*, the inoculated *N. benthamiana* seedlings were further subjected to RT-PCR identification of ACLSV, a PLPaV-co-infecting virus. The results showed all *N. benthamiana* seedlings to be infected by ACLSV, suggesting that PLPaV does not infect *N. benthamiana*. No symptoms were observed for inoculated seedlings of *P. sativum*, *S. lycopersicum*, *C. amaranticolor* and *C. quinoa*, but clear symptoms were observed in the remaining test plants (Table 1).

**Table 1:** Host range analysis of PLPaV of ten plants by mechanical inoculation with XJ-6 peach sap and indexed by RT-PCR or followed by nested PCR (nRT-PCR).

Family	Plant	Symptom^a^	Identification manner^b^	
			RT-PCR	nRT-PCR
*Fabaceae*	*Vigna unguiculata*	CS	0/8	6/8
	*Vicia faba*	CS	0/5	2/5
	*Pisum sativum*	no	4/5	4/5
*Solanaceae*	*Nicotiana occidentalis*	Dw, Ma	0/5	1/5
	*Nicotiana benthamiana*	Ma	0/5	0/5
	*Solanum lycopersicum*	no	0/6	2/6
*Cucurbitaceae*	*Cucurbita moschata*	Chl	0/5	3/5
	*Cucumis sativus*	CS	0/5	1/5
*Chenopodiaceae*	*Chenopodium amaranticolor*	no	0/8	2/8
	*Chenopodium quinoa*	no	0/8	1/8

^a^CS, chlorotic spot; Dw, dwarf; Ma, malformation; Chl, chlorosis; no, asymptomatic. ^b^Plants positive for PLPaV infection/total number of plants inoculated.

Next, 80 peach leaf samples were randomly collected from national (20 samples from Zhengzhou) and local (20 samples from Wuhan) peach germplasm nurseries and peach orchards (40 samples from Wuhan) for RT-PCR identification of PLPaV. Only three samples from two germplasm nurseries (two from Wuhan and one from Zhengzhou) were positive for the virus, suggesting a low incidence in the field. For each of the positive samples, two clones of the partial Hel gene obtained from the amplified bands were sequenced and aligned, and the results revealed high identity ranging from 97.92% to 100% among them but low identity, from 85.83% to 87.65%, with the PLPaV XJ-6 isolate (Fig. S6).

## Discussion

Smaller and cracked fruit disease affects peach, resulting in severe yield and quality losses; however, the responsible etiological agent has remained unknown to date. Deep sequencing of sRNA revealed three viruses (ACLSV, PBNSPaV and PLPaV) and two viroids (PLMVd and HSVd) in the leaves of an affected tree. Among them, ACLSV (genus *Trichovirus*, family *Betaflexiviridae*) is either latent or induces dark-green mottled patterns on the peach fruits^1^; PBNSPaV (genus *Ampelovirus*, family *Closteroviridae*) is associated with bark necrosis and stem-pitting disease in peach^17,18^. PLMVd (genus *Avsunviroid*, family *Avsunviroidae*) causes deformations and discoloration of fruits, which usually present cracked sutures and enlarged roundish stones^19^, and HSVd (genus *Hostuviroid*, family *Pospiviroidae*) is related to dappled peach fruit^20^. Therefore, these viruses and viroids provide no clues regarding the symptoms observed. However, following grafting of the GF305 peach seedlings using XJ-6 sample buds, smaller and cracked fruit symptoms did not appear on the fruits. Furthermore, five samples (from the 40 ones from Wuhan) showing smaller and cracked symptoms that were collected in the field and subjected to RT-PCR analysis did not exhibit the presence of PLPaV, suggesting that the symptoms were most likely not induced by the viral pathogens. Nonetheless, we cannot exclude the possibility that a viral pathogen is responsible for the symptoms, as symptoms observed in the field might become latent in GF305 seedlings due to changes in environmental factors and the peach varieties analyzed. In contrast, distinct from the symptoms of XJ-6 leaves in the field, a novel symptom characterized by leaf pitting appeared on three replicate seedlings (Fig. 1C). Correspondingly, these seedlings were all infected by PLPaV rather than by HSVd, ACLSV or PBNSPaV (Fig. S4). Additional symptoms of chlorosis, characterized by PLMVd infection, also appeared along leaf edges^16^. However, the biological features induced by the co-infected known viruses and viroids are well characterized, with no associated leaf-pitting symptoms. Thus, we conclude that PLPaV is most likely related to the novel symptoms, and Peach leaf pitting-associated virus is proposed as a novel virus. A final decision concerning PLPaV as being responsible for the peach leaf-pitting symptom requires fulfillment of Koch′s postulates. However, we attempted for two years to construct an infectious clone to inoculate seedlings but without success, as the cDNA of PLPaV RNA1 was shortened after cloning into a vector due to an as-yet-unknown reason.

PLPaV has a genomic organization similar to known fabaviruses (Fig. S3A), which, together with the phylogenetic analysis based on its RdRp, supports the identification of PLPaV as a novel fabavirus. Regardless, PLPaV exhibits many novel molecular features that distinguish it from other members of *Fabavirus*. For example, the first potential initiation codon of PLPaV RNA1 and PLPaV RNA2 is located in a residual context of (UGCA**AUG**G) and (UCAG**AUG**C), respectively, which are distinct from those of PrVF RNA1 (CCCA**AUG**G) and RNA2 (UUUC**AUG**C)^21^ as well as other fabavirus RNAs 1 and 2 (UAAA**AUG**G)^8^. Moreover, PLPaV only contains two putative signature sequence (AACAGCUUUC) repeats in 5′-UTRs, which is similar to the situation in PrVF (two repeats of AACCGCUUUC) but clearly fewer than in other *Fabavirus* members^21,22^, e.g., *Broad bean wilt virus* 1 (BBWV-1) and *Broad bean wilt virus* 2 (BBWV-2) contain four such motifs in their 5′-UTRs^8,23^. Furthermore, the polyadenylation signal (AAUAAA) for most eukaryotic mRNAs^11^ and present in BBWV-1 and BBWV-2 was absent from both UTRs of PLPaV. PLPaV represents the longest 3′-UTR (627 nt without counting a poly(A) tail) of any member of *Fabavirus*^8,21,23^, which are known to range from 81 nt (CuMMV) to 603 nt (LMMV). We also verified the content of the poly(A) tail, which has not been identified in other fabaviruses. We found that the length is considerably shorter (25–48 nt) than those of plant mRNAs, with lengths of 150–200 nt but longer than 22 nt, which has been characterized as a minimum poly(A) length required for efficient replication of *Bamboo mosaic potexvirus* RNA^24^. Notably, two cytosines were identified within the poly(A) region; this characteristic has not been previously detected in viral RNAs, and the function is currently unknown. With regard to PLPaV ORFs, most of the dipeptides (4/5) delimiting fused proteins were identical to those observed in PrVF but distinct from those of other fabaviruses or comoviruses (Fig. S3A). Moreover, the proposed Co-Pro of PLPaV is clearly larger (46 kDa) than those (35 to 39 kDa) of other fabaviruses, analogous to that of PrVF (43.6 kDa). Conversely, other proteins with a similar size are common to all known members^21^ (Fig. S3A). Collectively, these data suggest that PLPaV together with PrVF are more divergent in molecular features than other fabaviruses. In agreement with these diverse molecular features, the phylogenetic tree constructed based on RdRp also positioned PLPaV together with PrVF in a separate cluster from other fabavirus species (Fig. 1A).

More than 30 viruses belonging to ten genera of eight families are known to infect peach^25,26^, though no fabaviruses thus far. To our knowledge, this is the first report to describe a fabavirus infecting peach. Except for PrVF, fabaviruses in previous studies have all been isolated from herbaceous plants, and PLPaV represents the second case of a fabavirus also infecting a fruit tree and a *Rosaceae* plant^21^. A wide range of fabaviruses have been reported to infect dicotyledonous and monocotyledonous plants, including those belonging to six families: Aizoaceae, *Amaranthaceae*, *Chenopodiaceae*, *Fabaceae*, *Gentianaceae*, and *Solanaceae*^21,27–29^. In the present study, we tested the host range of PLPaV on most of these plants; however, the virus showed very low titer in most of the tested plants (except *P. sativum*). This result suggests that infection of these plants by the virus in the field should be very difficult, and therefore PLPaV might have a narrower host range than known fabaviruses. Moreover, there was clearly a low incidence compared with co-infecting viroids and viruses in China^17^, replicating at a very low titer in GF305 peach. Thus, we hypothesize that transmission of this isolate to peach does not have a long history. Overall, PLPaV showed novel molecular and biological features, indicating distinct evolutionary tracers separating it from known fabaviruses isolated from herbaceous plants.

Analysis of siRNAs derived from the PLPaV genome revealed that most are 21 and 22 nt in length, suggesting that their genesis was most likely mediated by DICER-like (DCL) enzymes 4 and 2^30–32^. This result coincides with those reported for most positive-strand RNA viruses, e.g., *Citrus tristeza virus*^33^, SMV^14^, and *Pepino mosaic virus*^34^. The mode of synthesis of other-sized siRNA, e.g. 16 nt, 28 and 30 nt long, is far less well understood. Bias at the 5′ terminus of PLPaV siRNAs indicated a complicated situation because it revealed both an association with the siRNA size and polarity, which are related to different AGOs^35–37^. It is surprising that an absolute bias for 27-, 28- and 30-nt was observed only for the negative strand, which, to our knowledge, has not been reported for other viruses. Moreover, the nucleotide bias at the 5′ terminus of PLPaV siRNAs differed from that other viruses; e.g., for SMV, A is most abundant at the 5′-end of 21- and 22-nt siRNAs^14^, whereas for PLPaV, U and G are the most abundant for positive and negative strands, respectively (Fig. 5). These results may suggest that different AGO-containing complexes preferably load variable siRNAs in a manner that is not only dependent on their size; however, we cannot exclude that this phenomenon might be due to technical effects (e.g., adapters and barcodes), RNA structure or strand polarity^38,39^.

In summary, we describe the first novel fabavirus infecting peach, which has several biological and molecular features that have not been reported for other fabaviruses. We believe that the discovery of this novel virus will aid further elucidation of the molecular and biological features of fabaviruses, providing clarification of the etiological agent responsible and assistance in selecting sources and implementing management practices for controlling viral diseases.

## Materials and Methods

### Sample preparation and deep sequencing

Leaves were collected in Wuhan, Hubei Province, China, in May 2013 from a peach tree (XJ-6) showing smaller and cracked fruit symptoms (Fig. 1A). The collected leaves were quickly frozen in liquid nitrogen, preserved in carbon dioxide ice blocks and shipped for 3–4 days to Biomarker Technologies Corporation, Beijing, China, for deep sequencing. One microgram total RNA was extracted, a unique adapter was added, RT-PCR was performed, and the product was purified by polyacrylamide gel electrophoresis (PAGE) for small RNA library construction and sequencing using an Illumina HiSeq^TM^ 2000 (Illumina, Inc., San Diego, California, USA) at Biomarker Technologies Corporation.

### Small RNA sequence processing, assembly and virus genome identification

Small RNA sequences were processed as previously described^40^. Briefly, raw Illumina sRNA reads were trimmed and cleaned by removing adaptor sequences, sequences shorter than 16 nt or longer than 30 nt, low-quality reads, poly(A), or N tags. The cleaned sRNAs were sorted into separate groups according to their length, counted, and indicated with a bar graph. The cleaned sRNAs were *de novo* assembled using Velvet with a k-mer value of 17^41^ and CAP3 with default values^42^. The siRNAs were selected by removing known noncoding RNAs (rRNAs, tRNAs, small nuclear RNAs, and small nucleolar RNAs, among others) obtained from RFAM (http://www.sanger.ac.uk/Software/Rfam/ftp.shtml) and host genomic RNAs from NCBI. The resulting final contigs were used to query the GenBank nt and nr database using the BLAST program^43^ and aligned to known virus and viroid genomes collected from the NCBI GenBank database using Bowtie software^44^. Only small RNA reads of sequences identical or complementary to viral genomic sequences within two mismatches were recognized as vsiRNAs, termed (+) and (−) polarity, respectively. To analyze terminal nucleotides, sRNAs or siRNAs were sorted into separate groups according to their length and polarity, counted, and indicated with a bar graph. The distribution of vsiRNAs along the viral genome were determined using Bowtie software, allowing up to three mismatches after removal of those corresponding to repeat elements, and the base coverage at each position of the contigs was derived based on the mpileup file generated from alignments using SAMtools under default parameters^45^.

### Validation and completion of viral genome sequences by Sanger sequencing

Sequence gaps between clones were determined by RT-PCR using specific primers designed based on the obtained cDNA sequences (Table S1). The 5′ and 3′ terminal sequences of the viral RNA were determined by rapid amplification of cDNA ends (RACE) with a kit (GeneRacer™ Core kit, Cat no. 45-0168, Lot no. 1362098. Invitrogen, Carlsbad, USA) following the manufacturer′s instructions. The authenticity of the siRNA-assembled viral genome sequences was validated by Sanger sequencing of the RT-PCR products covering the entire genome. The amplified PCR products were cloned into the pMD18-T vector (TaKaRa, Dalian, China) and transformed into competent cells of *Escherichia coli* DH5α. Sequencing was performed at Sangon Biotech (Shanghai) Co., Ltd, China, and each nucleotide was determined from at least three independent overlapping clones. The obtained clone sequences were assembled together using DNAMAN version 6.0 (Lynnon Biosoft Corporation, USA, http://www.lynon.com/).

### Sequence analysis

Sequence similarity searches were performed using National Center for Biotechnology Information (NCBI) databases with the BLAST program. Multiple alignments of nucleic and amino acid sequences were conducted using MAFFT version 6.85, as implemented at http://www.ebi.ac.uk/Tools/msa/mafft/ with default settings, except for refinement with 10 iterations. The resulting data were analyzed using GeneDoc software^46^. Identity analyses were conducted using the MegAlign program (version 5.00) with the ClustalW method (DNASTAR Inc.). A phylogenetic tree was constructed based on the RdRp gene of typical and selected members of *Comovirirdae*, as previously described^16^. Evolutionary history was inferred with MEGA 5.0^47^ using the neighbor-joining (NJ) method^48^. Evolutionary distances were computed using the JTT matrix-based method^49^ as units of the number of amino acid substitutions per site. Prediction of proteolytic cleavage was performed using the following website (http://www.cbs.dtu.dk/services/SignalP-3.0/) with default settings^50^. Secondary structures of the terminal sequences of RNAs were determined online (http://mfold.rna.albany.edu/?q=DINAMelt/Quickfold)^51^. ORFs were deduced using ORFfinder (https://www.ncbi.nlm.nih.gov/orffinder/) with default settings.

### Virus detection by RT-PCR

Total RNA was extracted from peach leaves using a previously described cetyltrimethylammonium bromide-based method^52^. The extracted RNAs were subjected to first-strand cDNA synthesis using Moloney Murine Leukemia Virus (M-MLV) reverse transcriptase (Promega Corp., Madison, WI, USA) with random hexamer primers (TaKaRa Biotechnology Corp., Dalian, China) at 37 °C for 1 h. Double-stranded DNA was amplified using *Taq* DNA polymerase (TaKaRa Biotechnology Corp., Dalian, China) with specific primers (Table S2) and the following reaction conditions: initial denaturation at 94 °C for 3 min, followed by 35 cycles of denaturation at 94 °C for 30 s, annealing at 60 °C (PLMVd and HSVd), 55 °C (ACLSV and PBNSPaV) or 56 °C (PLPaV) for 30 s, and extension at 72 °C for 45 s and a final extension at 72 °C for 10 min. PLPaV was detected by RT-PCR according to the above description using the primer pairs Fa1-F /Fab5′R1R followed by nested-PCR using the primer pairs Fa1-F /Fab5′R1Rn with an annealing temperature at 60 °C for 30 s or using the primer pairs Fa1-1 /Fab5′R1R followed by nested-PCR using the primer pairs Fa1-1 /Fab5′R1Rn with an annealing temperature at 55 °C for 30 s (Table S2).

PCR products were subjected to 1% agarose gel electrophoresis, purified using an agarose gel DNA purification kit and ligated into the pMD18-T vector (TaKaRa Biotechnology Corp., Dalian, China), followed by transformation into *E. coli* DH5α.

### Biological test, host range and incidence analysis

Biological tests were conducted by grafting XJ-6 peach buds onto GF305 seedlings (two years old) in three replicates in February 2014, with each seedling grafted with three buds. The inoculated seedlings were kept in a greenhouse under natural conditions for symptom development.

Host range analysis was conducted as previously described^28^. Briefly, the crude sap of XJ-6 peach leaves was inoculated mechanically into leaves of ten plant species in four families (Table 1). The experiment was conducted twice, and for each test, five to eight plants of each species were inoculated and maintained in a greenhouse (16 to 26 °C under a long-day period) and monitored for up to one month for symptom development. Inoculated plants were assayed for viral infection based on total RNAs extracted from newly developed leaves, as described above.

For an incidence survey, 80 peach leaf samples were randomly collected from national (20 samples from National Fruit Tree Germplasm Repository, Zhengzhou, Henan province, China) and local (20 samples from Peach Variety Nursery, Fruit and Tea Research Institute, Hubei Academy of Agricultural Sciences, Wuhan, Hubei province, China) peach germplasm nurseries and peach orchards (40 samples from Wuhan, Hubei province, China) for RT-PCR identification of PLPaV. The procedure was as described above using the primer pairs Fa1-1/Fab 5′R1R targeting the partial Hel gene region (Table S2). The PCR products were analyzed on 1% agarose gels.

### Data availability

Sequence data supporting the findings of this study have been deposited in GenBank under accession numbers KY867750 and KY867751 for RNA1 and RNA2 of PLPaV, respectively. The remaining data are available within the article and its Supplementary Information files and from the corresponding author upon request.

## Electronic supplementary material

Dataset 1
