# Author Correction: Deep sequencing reveals the first fabavirus infecting peach

**DOI:** 10.1038/s41598-018-23058-2

**Published:** 2018-03-22

**Authors:** Yan He, Li Cai, Lingling Zhou, Zuokun Yang, Ni Hong, Guoping Wang, Shifang Li, Wenxing Xu

**Affiliations:** 1 State Key Laboratory of Agricultural Microbiology, Wuhan, Hubei 430070 P.R. China; 2 College of Plant Science and Technology, Huazhong Agricultural University, Wuhan, Hubei 430070 P.R. China; 3 Key Lab of Plant Pathology of Hubei Province, Wuhan, Hubei 430070 P.R. China; 4 State Key Laboratory of Biology of Plant Diseases and Insect Pests, Institute of Plant Protection, Chinese Academy of Agricultural Sciences, Beijing, 100094 P.R. China

Correction to: *Scientific Reports* 10.1038/s41598-017-11743-7, published online 12 September 2017

This Article contains errors in Reference 19 which was incorrectly given as:

Flores, R. *et al*. *Citrus tristeza virus* p23: a unique protein mediating key virus-host interactions. *Front. Microbiol*. **98**, 1–9 (2013).

The correct reference is listed below:

Flores, R. *et al*. Peach latent mosaic viroid: not so latent. *Mol. Plant Pathol*. **7**, 209–221 (2006).

