# Supplementary material for: Deep sequencing reveals the first fabavirus infecting peach
Source: Sci Rep. 2017 Sep 12;7:11329. doi: 10.1038/s41598-017-11743-7 (PMC5595849; doi:10.1038/s41598-017-11743-7)
Supplement: Supplementary file 1 — Dataset 1 [file 41598_2017_11743_MOESM1_ESM.doc]

**Deep sequencing reveals the first fabavirus infecting peach**

Yan He^1,2,3#^, Li Cai^1,2,3#^, Lingling Zhou^1,2,3^, Zuokun Yang^1,2,3^, Ni Hong^1,2,3^, Guoping Wang^1,2,3^, Shifang Li^4^*, Wenxing Xu^1,2,3^*

^1^State Key Laboratory of Agricultural Microbiology, Wuhan, Hubei 430070, P. R. China; ^2^College of Plant Science and Technology, Huazhong Agricultural University, Wuhan, Hubei 430070, P. R. China; ^3^Key Lab of Plant Pathology of Hubei Province, Wuhan, Hubei 430070, P. R. China; ^4^State Key Laboratory of Biology of Plant Diseases and Insect Pests, Institute of Plant Protection, Chinese Academy of Agricultural Sciences, 100094, Beijing

^#^These authors contributed equally to this work.

*Corresponding author

WenXing Xu, Associate Professor, Plant Pathology ([xuwenxing@mail.hzau.edu.cn](mailto:xuwenxing@mail.hzau.edu.cn))

Phone: (86) 27-87287576; Fax: (86) 27-87384670;

Shifang Li, Professor, Plant Pathology (sfli@ippcaas.cn).

**Running title:** the first fabavirus infecting peach

**Keywords:** peach, deep sequencing, siRNA, Peach leaf pitting-associated virus, *Fabavirus*

**
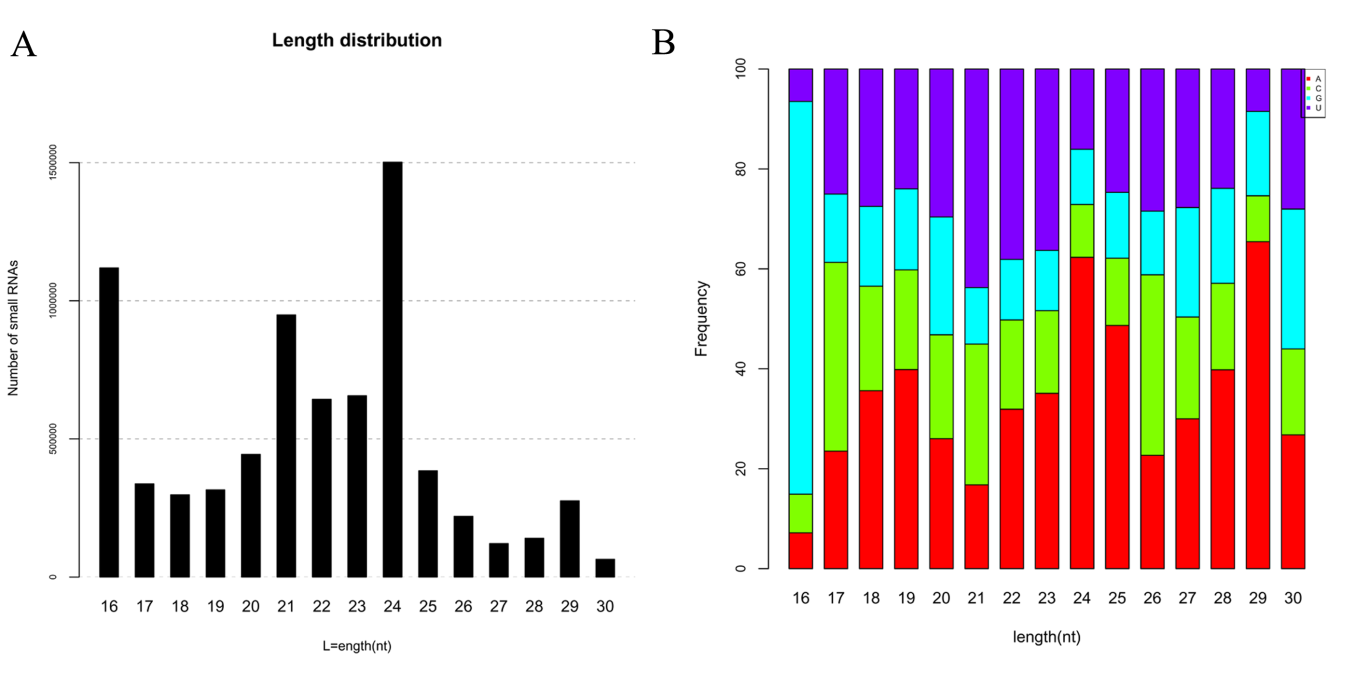
**

**Fig. S1** Size distribution and 5'-nucleotide frequency of total small RNAs (sRNAs) in the library prepared from XJ-6 peach leaves. (A) Bar graph of the sRNA read numbers in sizes from 16 to 30 nt. (B) Bar graph of the 5'-nucleotide frequencies of the sRNA reads in sizes from 16 to 30 nt.


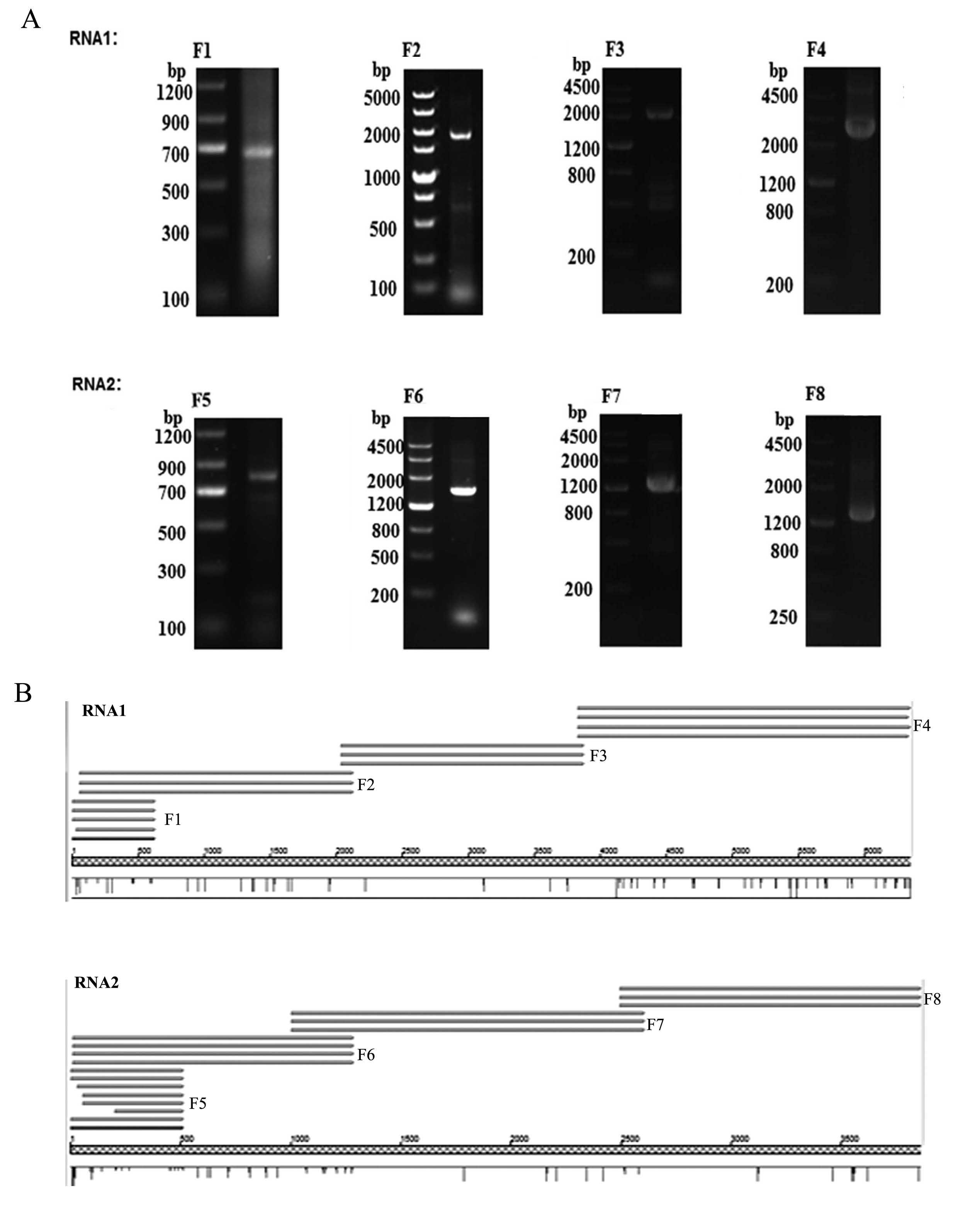


**Fig. S2** Agarose electrophoresis analysis of the RT-PCR products of the contigs covering the full genome of Peach leaf pitting-associated virus (PLPaV), and schematic diagram of assemble of the contigs. (A) RT-PCR products of the contigs F1 to F4 (RNA1) and F5 to F8 (RNA2) using the primers listed in Table S1. (B) Assemble of clone sequences that were randomly cloned and sequenced, covering most of the RNAs 1 and 2 strands (RNA1 and RNA2), using program DNaman version 6.0 (Lynnon Biosoft Corporation, USA, http://www.lynon.com/).


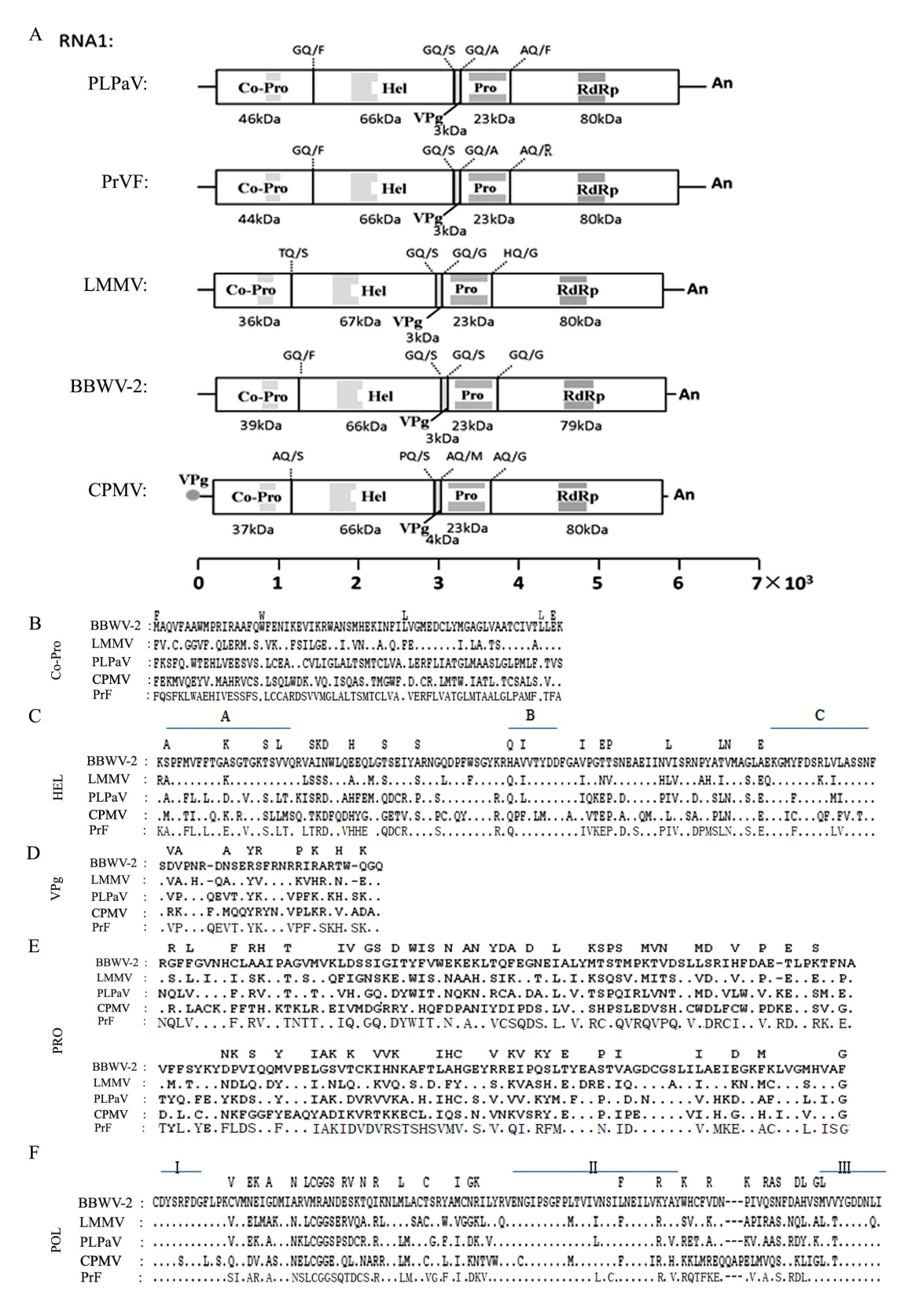


**Fig. S3** Schematic diagram of genetic organization of RNA-1 of PLPaV, (PrVF), *Lamium mild mosaic virus* (LMMV), *Broad bean wilt virus* 2 (BBWV-2) and *Cowpea mosaic virus* (CPMV), and the motifs conserved in their deduced proteins. (A) Open bars represent open reading frames (ORFs). The possible polyprotein cleavage sites are indicated by vertical lines showing the corresponding amino acids. The sizes of the mature proteins are indicated. (B-F) The motifs conserved in putative proteinase cofactor (Co-Pro), helicase (Hel), genome-linked protein (VPg), proteinase (Pro), and RNA-dependent RNA polymerase (RdRp), respectively. Dashes represent gaps between conserved regions.

**
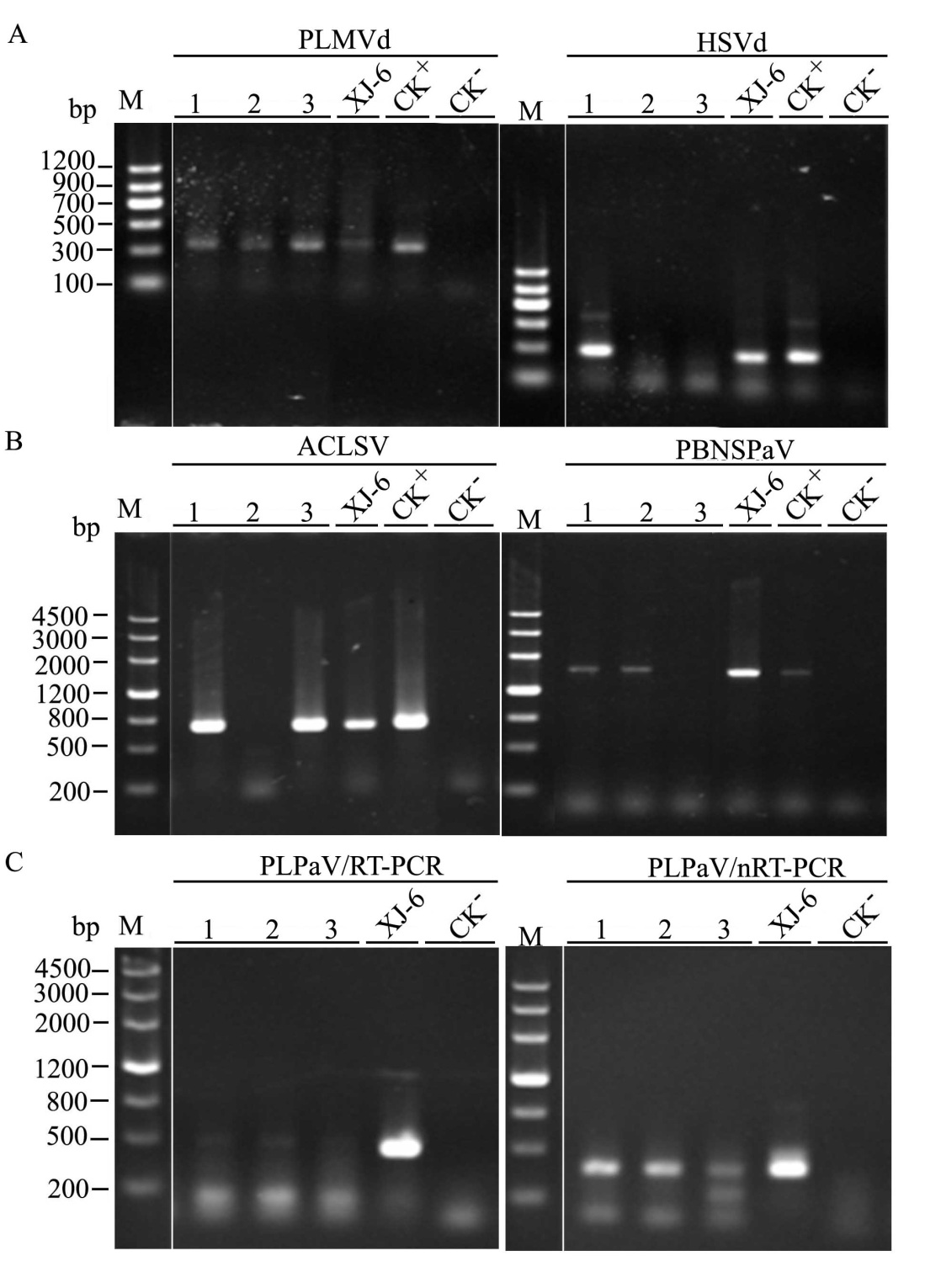
**

**Fig. S4** Agarose electrophoresis analysis of RT-PCR or nested RT-PCR (nest-RT-PCR) detection of viruses and viroids in three GF305 seedlings grafted with XJ-6 peach buds. (A and B) RT-PCR analysis of *Peach latent mosaic viroid* (PLMVd) and *Hop stunt viroid* (HSVd) (A), and of *Apple chlorotic leaf spot trichovirus* (ACLSV) and *Plum bark necrosis stem pitting-associated virus* (PBNSPaV) (B), respectively. (C) RT-PCR (left) and nest-RT-PCR (right) analysis of PLPaV, respectively. The primers involved in the detection are listed in Tables S2. M, marker; the numbers 1, 2 and 3 over the gels indicate the samples nos. 1, 2 and 3 GF305 peach seedlings grafted with XJ-6 peach buds; XJ-6, XJ-6 peach; CK^+^, plants infected with the target virus or virods; CK^-^, negative control of GF305 peach seedlings without inoculation of viruses or viroids.

**
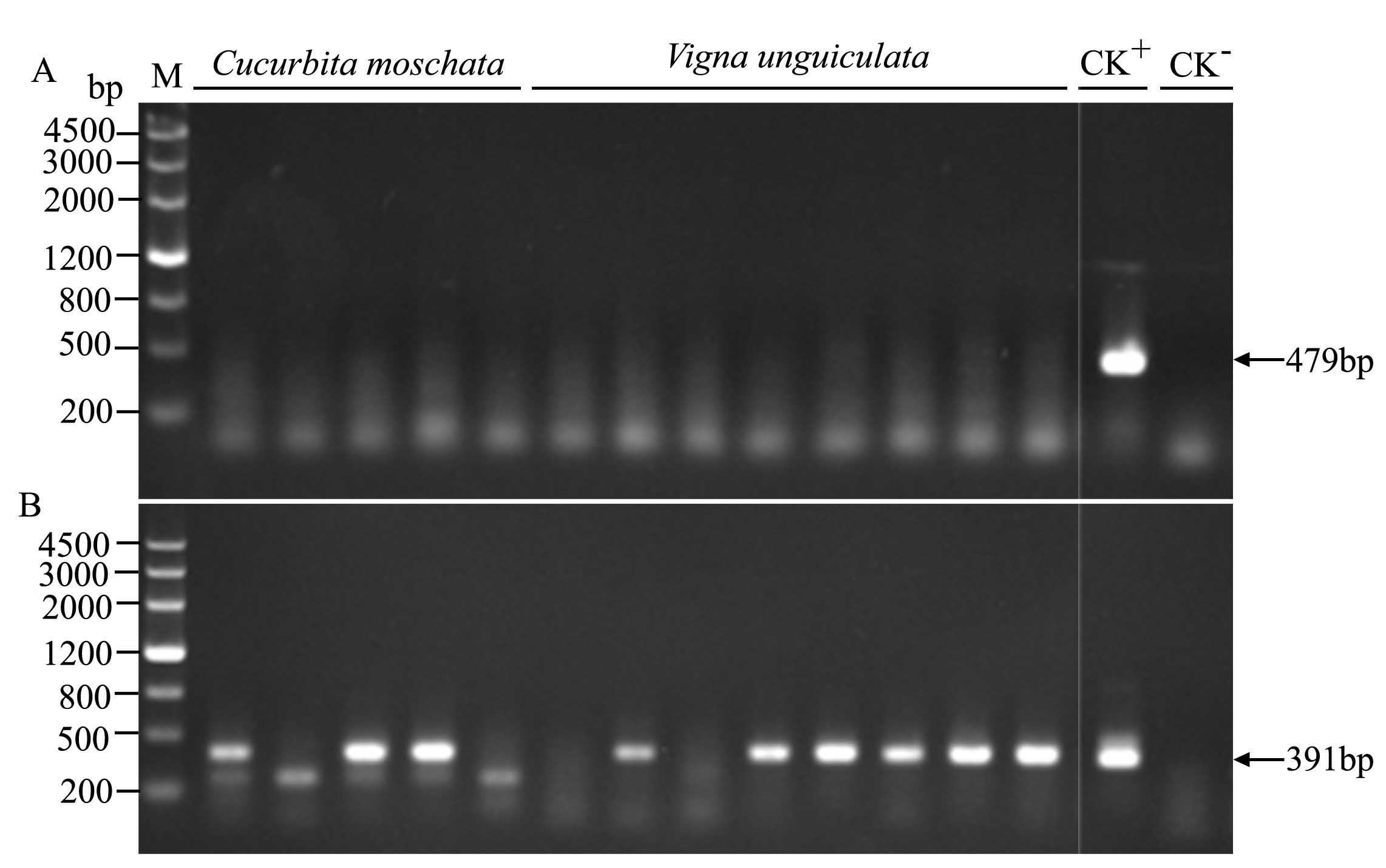
**

**Fig. S5** Representative RT-PCR and nRT-PCR analysis of PLPaV in plants mechanically inoculated with saps of XJ-6 peach leaves at 20 dpi. (A and B) RT-PCR (above) and nRT-PCR (below) analysis of PLPaV in five and eight seedlings of *Cucurbita moschata* and *Vigna unguiculata*, respectively. The primers involved in the detection are the primer pairs (Fa1-1 /Fab5'R1R) or following by nest-PCR using the primer pairs (Fa1-1 /Fab5'R1Rn) (Tables S2). M, marker; the symbols ‘CK^+^’ and ‘CK^-^’ over the gels indicate the samples of XJ-6 peach and the negative control of the plants without inoculation of viruses or viroids.

**
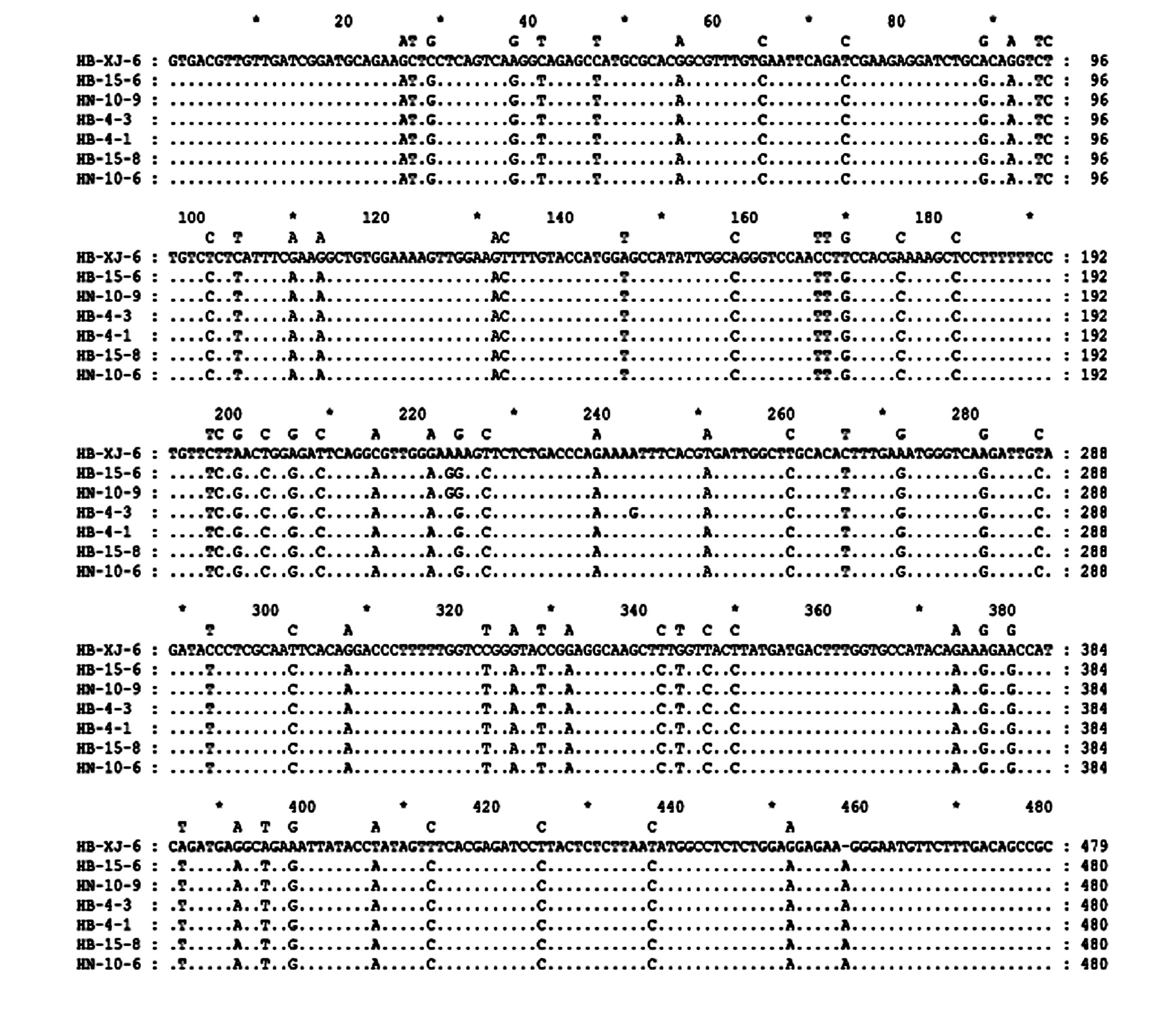
**

**Fig. S6** Sequences alignment of partial HEL sequences of PLPaV isolates. HN- and HB-, the isolates from Henan and Hubei province, respectively. The accession numbers for the partial HEL sequences are MF589708 (HB-15-6 and HN-10-9), MF589709 (HB-4-3) and MF589710 (HB-4-1, HB-15-8 and HN-10-6).

| **Primer name** | **Primer sequences**  **(5'-3')** | **Denaturing temperature (℃)** | **Length**  **(bp)** | **Targeted fragment** |
| --- | --- | --- | --- | --- |
| GeneRace™ 5'n | GGACACTGACATGGACTGAAGGAGTA | 62 | 632 | F1 |
| 5'R1Rn | GCTCAACCTCAGAGGAATCTTGC |  |  |  |
| Fab5'R1F | AAATATTAAAACAAACAGCTTTCGTT | 52 | 2100 | F2 |
| Fab5R1Fn | TCATCTGATGGTTCTTTCTGTATGGC |  |  |  |
| Fab1-4F | TAGATACCCTCGCAATTCACAG | 58 | 1849 | F3 |
| Fab1-4R | TTCCGGCATGAAACTAGCAC |  |  |  |
| GeneRace™ 3'n | CGCTACGTAACGGCATGACAGTG | 60 | 2471 | F4 |
| Fab3'R1Fn | TTGCACATAGCAGGAAATGGGACA |  |  |  |
| GeneRace™5'n | GGACACTGACATGGACTGAAGGAGTA | 65 | 513 | F5 |
| 5'R2Rn | TTGGCCATAAGCTCCACGCAACCTC |  |  |  |
| Fab5'R1F | AAATATTAAAACAAACAGCTTTCGTT | 52 | 1286 | F6 |
| Fab5R2Fn | CCTCTCTGGTCCATGTATCACACG |  |  |  |
| Fab3-2F | ATTTCCAATAGACCAGCACGAG | 58 | 1606 | F7 |
| Fab3-2R | CTGATAAGGGAAAACCCGCACT |  |  |  |
| GeneRace™ 3'n | CGCTACGTAACGGCATGACAGTG | 60 | 1369 | F8 |
| Fab3'R2Fn | GGGTTCGTGACTCTGTAAACTCAGC |  |  |  |

**Table S1 Primers used for cloning full-length of PLPaV**

**Table S2 Primers used for detection of PLMVd, HSVd, ACLSV, PBNSPaV and PLPaV**

| | **Primer name** | **Primer sequences （5'-3'）** | **^*^T (℃)** | **Length (bp)** | **Targeting regions** |  | **Reference** | | --- | --- | --- | --- | --- | --- | --- | | PLMVd Pr1 | ATCACACCCCCCTCGGAACCAA | 60 | 337 | Full length |  |  | | PLMVd Pr2 | CCAGGTACCGCCGTAGAAACTG |  |  |  |  |  | | HSVd-P1 | TTATGCCACCATGATAACACC | 60 | 370 | Full length |  | This study | | HSVd-P2 | GACTTCATCACCTTAGCGACA |  |  |  |  |  | | ACLSV-216F | GGTGAGAGGCTCTATTCACATCTTG | 55 | 261 | RNA polymerase |  |  | | ACLSV-216R | GGAGCTTTTCACCCCAGCAATTGG |  |  |  |  |  | | PBNSPaV-HSP-F | GGAATTGACTTCGGTACAAC | 55 | 195 | Hot Shock Protein |  |  | | PBNSPaV-HSP-R | TTCGGTGGTGGTACTTTCG |  |  |  |  |  | | Fa1-F | GATCGGATGCAGAAGCTCCTC | 60 | 468 | Helicase |  | This study | | Fab5‘R1R | GCGGCTGTCAAAGAACATTCCC |  |  |  |  |  | | Fab5'R1Rn | TCATCTGATGGTTCTTTCTGTATGGC | 60 | 380 |  |  | This study | | Fa1-1 | GTGACGTTGTTGATCGGATGC | 55 | 480 | Helicase |  | This study | | Fab5'R1R | GCGGCTGTCAAAGAACATTCCC |  |  |  |  |  | | Fab5'R1Rn | TCATCTGATGGTTCTTTCTGTATGGC | 55 | 391 |  |  | This study | |
| --- | --- | --- | --- | --- | --- | --- | --- | --- | --- | --- | --- | --- | --- | --- | --- | --- | --- | --- | --- | --- | --- | --- | --- | --- | --- | --- | --- | --- | --- | --- | --- | --- | --- | --- | --- | --- | --- | --- | --- | --- | --- | --- | --- | --- | --- | --- | --- | --- | --- | --- | --- | --- | --- | --- | --- | --- | --- | --- | --- | --- | --- | --- | --- | --- | --- | --- | --- | --- | --- | --- | --- | --- | --- | --- | --- | --- | --- | --- | --- | --- | --- | --- | --- | --- | --- | --- | --- | --- | --- | --- | --- | --- | --- | --- | --- | --- | --- | --- | --- | --- | --- | --- | --- | --- | --- |

^*^ Denaturing Temperature.

Cui, H. G., Hong, N., Xu, W., Zhou, J. F. and Wang, G. (2011) First report of Plum bark necrosis stem pitting-associated virus in Stone Fruit Trees in China. *Plant Disease,* **95,** 1483.

Hernández, C. and Flores, R. (1992) Plus and minus RNAs of peach latent mosaic viroid self-cleave in vitro via hammerhead structures. *Proc. Natl. Acad. Sci. U.S.A.,* **89,** 3711-3715.

Zhu, H., Wang, G., Hu, H., Tian, R. and Hong, N. ( 2014) The genome sequences of three isolates of Apple chlorotic leaf spot virus from pear (*Pyrus* sp.) in China. *Canadian Journal of Plant Pathology* **36**.

**Table S3 Information of the viruses used for phylogenetic analysis.**

| **Genus** | **Species** | **Abbreviation** | Isolate | GenBank Acc. no. | | |
| --- | --- | --- | --- | --- | --- | --- |
|  |  |  |  | RNA1 | RNA2 | RdRp |
| ***Fabavirus*** | Prunus virus F | PrVF | 8816-v2 | KX269866 | KX269871 | ANH71248 |
|  | *Broad bean wilt virus 1* | BBWV-1 | Ben | AY781171 | AY781172 | AAX12375 |
|  | *Broad bean wilt virus 2* | BBWV-2 | IP | AB023484 | AB018698 | BAB40439 |
|  | *Mikania micrantha mosaic virus* | MMMV | GZ1 | NC_011190 | NC_011189 | YP_002158824 |
|  | *Gentian mosaic virus* | GeMV | N-1 | AB084452 | AB084453 | BAD99001 |
|  | *Cucurbit mild mosaic virus* | CuMMV | Beijing | EU881936 | EU881937 | ACK76423 |
|  | *Lamium mild mosaic virus* | LMMV | PV-0454 | NC_023016 | NC_023017 | YP_008877634 |
| ***Comovirus*** | *Squash mosaic virus* | SqMV | Y-SqMV | NC_003799 | NC_003800 | NP_620657 |
|  | *Cowpea severe mosaic virus* | CPSMV | / | NC_003545 | NC_003544 | NP_619518 |
|  | *Bean pod mottle virus* | BPMV | KY G-7 | NC_003496 | C_003495 | NP_612349 |
|  | *Cowpea mosaic virus* | CPMV | / | NC_003549 | NC_003550 | NP_613283 |
|  | *Red clover mottle virus* | RCMV | S | X64886 |  | CAA46104 |
| ***Nepovirus*** | *Arabis mosaic virus* | ArMV | NW | NC_006057 | NC_006056 | YP_053925 |
|  | *Grapevine fanleaf virus* | GFLV | SACH44 | KC900162 | KC900163 | AGT42200 |
|  | *Grapevine deformation virus* | GDefV | / | NC_017939 | NC_017938 | YP_006347591 |
|  | *Beet ringspot virus* | BRSV | / | NC_003693 | NC_003694 | NP_620112 |
|  | *Tomato black ring virus* | TBRV | MJ | NC_004439 | NC_004440 | NP_958814 |
| ***Sadwavirus*** | *Satsuma dwarf virus* | SDV | S-58 | AB009958 | AB009959 | BAA76746 |

“/” refers to no designated isolates.
